# *Geobacter* Dominates the Inner Layers of a Stratified Biofilm on a Fluidized Anode During Brewery Wastewater Treatment

**DOI:** 10.3389/fmicb.2018.00378

**Published:** 2018-03-06

**Authors:** Sara Tejedor-Sanz, Patricia Fernández-Labrador, Steven Hart, Cesar I. Torres, Abraham Esteve-Núñez

**Affiliations:** ^1^Department of Chemical Engineering, University of Alcalá, Alcalá de Henares, Spain; ^2^IMDEA Water Institute, Alcalá de Henares, Spain; ^3^Mahou San Miguel, Madrid, Spain; ^4^Biodesign Swette Center for Environmental Biotechnology, Arizona State University, Tempe, AZ, United States; ^5^School for Engineering of Matter, Transport and Energy, Arizona State University, Tempe, AZ, United States

**Keywords:** *Geobacter*, fluidized bed, bioelectrochemistry, microbial stratification, wastewater treatment, microbial electron transport, microbial electrochemical technologies

## Abstract

In this study, we designed a microbial electrochemical fluidized bed reactor (ME-FBR), with an electroconductive anodic bed made of activated carbon particles for treating a brewery wastewater. Under a batch operating mode, acetate and propionate consumption rates were 13-fold and 2.4-fold higher, respectively, when the fluidized anode was polarized (0.2 V) with respect to open circuit conditions. Operating in a continuous mode, this system could effectively treat the brewery effluent at organic loading rates (OLR) over 1.7 kg m^-3^_NRV_ d^-1^ and with removal efficiencies of 95 ± 1.4% (hydraulic retention time of 1 day and an influent of 1.7 g-COD L^-1^). The coulombic efficiency values highly depended upon the OLR applied, and varied from a 56 ± 15% to 10 ± 1%. Fluorescence *in situ* hybridization (FISH) analysis revealed a relative high abundance of *Geobacter* species (*ca*. 20%), and clearly showed a natural microbial stratification. Interestingly, the *Geobacter* cluster was highly enriched in the innermost layers of the biofilm (thickness of 10 μm), which were in contact with the electroconductive particles of bed, whereas the rest of bacteria were located in the outermost layers. To our knowledge, this is the first time that such a clear microbial stratification has been observed on an anode-respiring biofilm. Our results revealed the relevant role of *Geobacter* in switching between the electrode and other microbial communities performing metabolic reactions in the outermost environment of the biofilm.

## Introduction

Water purification technologies based on biological processes require a suitable electron acceptor to consume the electrons generated in the oxidation of organic waste. In this context, microbial electrochemical technologies (METs) represent a promising field based on the effective redox coupling between microbial metabolism and electrically conductive materials (Du et al., 2007).

Although urban wastewater (Min and Logan, 2004; Brown et al., 2015) has been the most common biodegradable fuel tested in METs, industrial organic matter sources such as food industry residues have been extensively tested in the last decade (Cercado-Quezada et al., 2010; Kelly and He, 2014; Çetinkaya et al., 2015). From the very beginning of research in this field, brewery wastewater has received much attention since the organic components in brewery effluents (consisting of sugars, soluble starch, ethanol and volatile fatty acids) are highly biodegradable (Feng et al., 2008; Dong et al., 2015).

A potential advantage of bioelectrochemical systems is the higher resistance of electrogens to disturbances caused by shocks in the organic loading when compared to methanogens (Kaur et al., 2014). Anaerobic digestion has been typically the technology used by brewery plants for eliminating the organic matter of its effluents. One of the problematic factors of anaerobic digesters is the slow growth and the high sensitivity of methanogens to a wide variety of inhibitory compounds (Chen et al., 2008). This can lead to an accumulation of the volatile fatty acids (VFAs) (acetic and propionic acids principally) and a pH drop (Franke-Whittle et al., 2014) if the feeding presents disturbances like organic load shocks. In this regard, METs have been shown to be systems in which VFAs can be rapidly consumed by electrogens under the presence of an anode acting as electron acceptor (Asensio et al., 2016; Cerrillo et al., 2016).

The capacity of biological treatment systems is determined by the biomass amount (concentration, volume, etc.) and its activity. One of the engineering designs that have allowed one to optimize the mix of these two variables is the fluidized bed reactor. This configuration has been merged with a MET by using an electrically conductive fluid-like bed, resulting in a microbial electrochemical fluidized bed reactor (ME-FBR). In this system, the fluidized bed functions as a fluid-like polarized three-dimensional electrode with large specific surface to stimulate the degradation of organic matter by microbial electrogenesis. Interestingly, direct extracellular electron transfer between *Geobacter* planktonic cells and a fluidized anode has been reported in a ME-FBR (Tejedor-Sanz et al., 2017a). This fact suggests a new paradigm in bioelectrochemical systems in which direct extracellular electron transfer (EET) does not proceed through biofilm formation. In contrast to this planktonic interaction, by using porous particles in motion and with a more hydrophilic and irregular surface than glassy carbon beads (i.e., activated carbon), a biofilm architecture of a mixed culture can also be achieved (Kong et al., 2011; Deeke et al., 2015; Tejedor-Sanz et al., 2017b).

Electroactive bacteria commonly interact with electrodes directly by forming a biofilm. Techniques based on sequencing the 16S rRNA gene have allowed one to identify the main microbial communities enriched in anode-reducing biofilms. Actually, *Geobacter* species have been reported to dominate the microbial communities found in anodes composed of mixed populations (Kan et al., 2011; Kiely et al., 2011; Yates et al., 2012). This genus has also been identified in the granules of an upflow anaerobic sludge blanket (UASB) reactor treating brewery waste (Shrestha et al., 2014). In spite of the absence of an electrode, *Geobacter* was found to perform direct extracellular electron transfer (DEET) by exchanging the electrons with methanogenic communities, through direct interspecies electron transfer (DIET). Specifically, *Methanosarcina barkeri* has been shown to be capable of performing DIET in co-cultures with *Geobacter* species (Rotaru et al., 2014, 2015). DIET can also take place with a mineral as a mediator, a process in which different species use as conduits of electrons nano-mineral particles or conductive surfaces such as activated carbon granules or biochar (Kato et al., 2012; Liu et al., 2012). This phenomena has also been described to stimulate methane production and *Geobacter* growth (Cruz Viggi et al., 2014; Shrestha and Rotaru, 2014; Li et al., 2015). All these findings suggest that co-aggregation of *Geobacter* species and methanogens may be a common phenomenon in methanogenic environments and that might be relevant with respect to methane production in anaerobic digesters. Thus, besides the engineering aspects, the study of the microbial diversity and their interactions in these reactors can provide potential tools for optimizing its performance.

In our work we aim to characterize a ME-FBR as a technology for removing the organic matter from a brewery wastewater. This effluent contains both fermentable and non-fermentable matter, and thus promotes the proliferation of a wide range of microbial communities that we aim to characterize. In addition to studying the performance of the system, we investigate the anodic biofilm structure developed on the fluidized electrode in order to provide insights into the interspecies interactions in these electroactive biofilms.

## Materials and Methods

### Wastewater Description and Analysis

The wastewater samples used for the all experiments were collected from the brewery plant Mahou-San Miguel in Alovera, Guadalajara, and were frozen at -20°C until used. Wastewater samples were taken from the homogenization tank that fed the anaerobic digester of their brewery wastewater treatment plant (WWTP). This water came from a previous coagulation treatment and pH-adjustment. When a batch was used as influent, this was stored in a fridge at 4°C. A complete analysis (physical -chemical parameters) of the wastewater was performed for the first batch of wastewater collected at the treatment plant. For the remaining samples, only the total chemical oxygen demand (COD) was analyzed.

Effluent samples were frozen prior to its analysis (-20°C). COD was measured with kits from Merck Millipore (Germany) by adding 3 ml of the sample to kit preparations. The samples tubes were digested for 2 h at 148°C in a Merck Spectroquant^®^ TR420 and determined by a Merck Spectroquant^®^ NOVA60 photometer. The volatile fatty acids were measured in a gas chromatograph (Bruker 430 GC), equipped with a fused silica capillary column of 30 mm × 0.25 mm × 0.25 μm and a conductivity detector. The measurements were performed at a detector temperature of 200°C, 240°C for the injector, and 180°C for the oven, and nitrogen as mobile phase (1 mL min^-1^). Methane was detected on a Varian 3350 chromatograph equipped with a packed column (Porapack N 80/100) and a TCD detector. Nitrogen was used as the carrier gas (20 mL min^-1^). The column was set to 80°C, the injector to 110°C and the detector to 200°C.

### Experimental Set-up

The ME-FBR was designed as previously described (Tejedor-Sanz et al., 2017b). A schematic of the system is shown in Supplementary Figure 1. At the top of the column the reactor was closed with a rubber tap in order to maintain an anoxic environment. For fluidizing, a recirculation flow was drawn from the top section using a peristaltic pump (Heidolph 5006, Germany). The total volume of the reactor was 0.68 L (including the recirculation tube and the bed volume). The anode consisted of a bed composed of 80 mL (43 g) Aquasorb activated carbon particles (0.6–1 mm diameter) from Chemviron Carbon (Belgium). A graphite plate (20 × 80 mm) that was vertically immersed in the fluidized bed was used as the current collector. The cathode consisted of a graphite felt plate (100 × 70 × 6 mm, RGV 2000) from Mersen (France). A Ag/AgCl 3 M KCl electrode (HANNA) was employed as reference electrode. The ME-FBR was operated as a three-electrode electrochemical cell and the conductive bed worked as the anode by polarizing at 0.2 V (all potentials are reported versus Ag/AgCl electrode). The potentiostat used was a NEV3 Nanoelectra (Spain). The electrolyte velocity used during all the experimental period was of 0.68 cm s^-1^.

A non-electrochemical fluidized bed reactor (biolite M-FBR) was constructed with the same design as the ME-FBR but without electrodes and a bed of 80 mL of biolite particles (particle diameter = 0.25–0.32 mm, ρ = 1.25 kg L^-1^, specific surface area = 0.6 m^2^ g^-1^). The electrolyte velocity used for this reactor was 0.34 cm s^-1^. This configuration acted as a non-bioelectrochemical control.

Each of these reactors was inoculated with 50 mL of the settled sludge from an urban WWTP. This sludge was a mix of liquor from the activated sludge reactor and denitrifying pond of the urban WWTP. For the acclimation of the biomass, the systems were previously operated under batch mode by feeding acetate supplemented brewery wastewater mixed with buffered medium (*ca.* during 20 days) (0.5 g L^-1^ of NH_4_Cl, 0.6 g L^-1^ NaH_2_PO_4_⋅6H_2_O, 0.1 g L^-1^ and KCl, 10 mL L^-1^ and bicarbonate from 50 to 75 mM. After *ca.* 1 month, these reactors were operated in a continuous mode [starting at hydraulic retention timecdot (HRT) of 2.4 days] with increasing loads of brewery wastewater added as influent, and decreasing concentrations of the bicarbonate buffer.

A peristaltic pump (Watson and Marlow 205S, United States) was used for continuously feeding the reactors from the bottom of the column. The effluent outlet was located at the upper part of the column.

### Experimental Procedures

#### Assays Feeding Real Wastewater at Continuous Mode

**Table 1** summarizes the operating conditions for each experimental period and the duration of these conditions for the ME-FBR. The M-FBR was operated in parallel from day 1 to day 119 at 5 different OLR conditions. The organic loading rate (OLR) was varied either by supplying a wastewater with different COD concentrations or by increasing the influent flow rate (varying HRT). COD, VFAs and current production were measured in this experimental phase.

**Table 1:** Reactor operating conditions during the continuous mode period.

Duration (days)	COD influent (mg L^-1^) (*n* = 3)	HRT (h)	OLR (kg COD m^-3^_NRV_ d^-1^)	Feed
1–24	580 ± 30	53	0.24 ± 0.01	BWW + 75 mM NaHCO_3_
25–55	870 ± 50	53	0.36 ± 0.02	BWW + 50 mM NaHCO_3_
55–89	1480 ± 97	53	0.62 ± 0.04	BWW
90–111	900 ± 5	53	0.38 ± 0.01	BWW
113–129	610 ± 49	53	0.25 ± 0.02	BWW
130–157	2750 ± 84	53	1.15 ± 0.04	BWW
142–159	1600 ± 20	28	1.26 ± 001	BWW
160–168	1690 ± 105	21	1.73 ± 0.11	BWW

An open circuit control (OC) assay with the ME-FBR was performed by running this reactor at an OLR of 1.46 ± 0.2 kg COD m^-3^_reactor_ d^-1^ (electrodes disconnected, no current flow). Subsequently, the ME-FBR was polarized back to 0.4 V in order to compare the COD removal under both conditions.

Samples of the effluents were collected daily in order to measure the total COD.

#### VFAs Degradation Tests

For further analyzing the VFAs degradations, the individual removal of acetate (10.6 ± 2.8 mM) and propionate (5.3 ± 0.2 mM) in the ME-FBR was analyzed using a synthetic media. The medium for this assay contained salts as described before and bicarbonate buffer (50 mM). The two acids were individually and alternatively added to the ME-FBR in batch mode, first with the electrodes polarized (E_fluidized anode_ = 0.2 V), and subsequently, with the system at OC conditions. Samples were taken periodically and each test was performed three times.

### Calculations and Data Analysis

The linear velocity of the recirculating electrolyte was calculated with the flow rate of the recirculation pump (L min^-1^) and the column internal diameter (46 mm) (flow rate/column section). The coulombic efficiency (CE) values were calculated by dividing the daily harvested charge in the conductive bed (Q_i_) by the theoretical charge that could be harvested from the total organic matter (COD) removed each day. Q_i_ was obtained at each assay from the integration of the chronoamperometric curves (current response over time). Q_t_ was calculated by estimating the total charge contained in all of the COD removed. For this calculation, we considered that 4 electrons are exchanged per mol of oxygen consumed to theoretically oxidize the organic matter. Current density values and OLR are given per net volume of reactor (NRV), which was of 0.6 L.

COD removal, CE, organic removal rates (ORR) and current density values correspond to averages obtained from the samples collected after the reactor reached a stationary state (after operating for > 2-fold the HRT). A pair sample *t*-test analysis was performed in order to compare both the COD removal (%) and the current density production enhancement as the organic loading rate was increased (significance level of 0.05). OriginPro 8 SR0 hypothesis testing tool was used for this analysis.

### Scanning Electron Microscopy Examination

Scanning electron microscopy (SEM) was used to study the microbial colonization of the activated carbon particles in the ME-FBR. The collected samples were gently rinsed with buffered medium prior to its preparation. Samples were fixed with 5% (v/v) glutaraldehyde in cacodylate buffer (0.2 M, pH 7.2) and dehydrated through a graded series of ethanol solutions (25, 50, 70, 90, and 100%; 10 min each stage). Subsequently, the samples were rinsed two times in acetone for 10 min and immersed in anhydrous acetone at 4°C overnight. Finally, the samples were dried in CO_2_ at the critical point and coated with gold (50 nm thickness) using a Polaron E5400 coating system and argon as a process gas. Micrographs were taken using a scanning electron microscope DSM-950 (Zeiss).

### FISH Analysis

The microbial community was analyzed with probe combinations (50 ng μL^-1^) of either (i) Cy5-ARC915 to target most Archaea (Stahl and Amann, 1991), Cy3-EUB338 or Cy5-EUB338 to target most bacteria (Amann et al., 1990) and (ii) Fluo-GEO3-A, Fluo-GEO3-B and Fluo-GEO3-A to target *Geobacter* genera (Richter et al., 2007), with the helpers HGEO3-4 and HGEO3-4. As a negative control, we employed a non-specific probe (non-338) with fluorescein as fluorochrome (see Supplementary Figure 7) in order to control non-specific binding of EUB 338. In addition, as a second negative control we hybridized, but without probes, activated carbon particles without biofilm in order to discard any possible autofluorescence. At least 2 particles were hybridized for each of the following independent combinations: (1) particle with non-338; (2) abiotic particle with hybridization buffer; (3) and (4) particle with Eubacteria + *Geobacter* probes + DAPI; (5) particle with Eubacteria + Archea probes + DAPI; (6) particle with *Geobacter* probe + DAPI. Probes were synthesized by IDT (Coralville, IA). DAPI (4′,6-diamidino-2-phenylindole) was used to stain all nucleic acids in order to quantify all of the attached biomass. At the time of the collecting the fluidized particles, the ME-FBR was operating at an OLR of 1.7 kg-COD m^-3^_reactor_ d^-1^. The hybridization protocol is described in the Supplementary Information. Images were taken in a Leica TCS SP5 AOBS Spectral Confocal Microscope, equipped with four laser (405, Ar, Kr/Ar, and He/Ne) (405, 561, and 633) and 4 prism spectrophotometer detectors. The objective used was a HCX APO L U-V-I 40.0x0.80 (water). The software was the Leica Confocal Software (LCS) for multi-dimensional image series acquisition. The software built 3D images from sequences of 18 images each one with an interval of 2 μm. Images were processed and analyzed using the software ImageJ 2.0.0 (further details described at Supplementary Information).

## Results and Discussion

**FIGURE 1:**
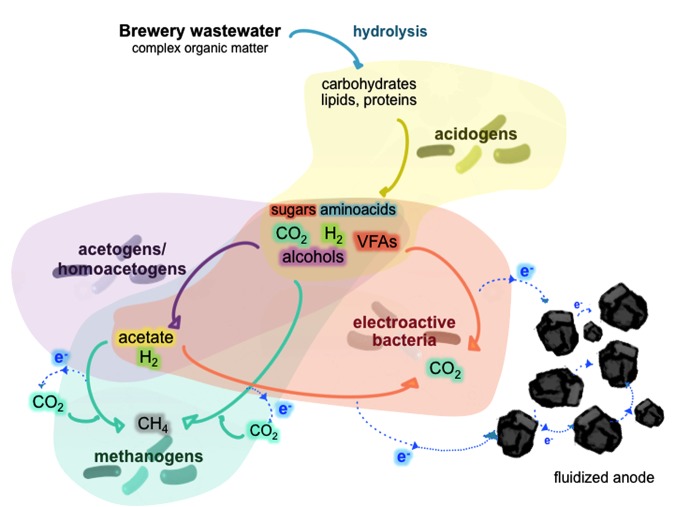
Schematic of the different anaerobic communities that might coexist within the ME-FBR and the possible competitive reactions among them for the electron donors.

The general characteristics of the brewery wastewater are presented in Supplementary Table 1. Since the wastewater came from a previous coagulation pretreatment, low amounts of suspended solids and nutrients (N and P) were found in this effluent (*ca*. 25 and 6.8 mg L^-1^, respectively). Nitrate and nitrite were below the detection limits, indicating that most of the nitrogen in the wastewater was in the form of ammonia and insoluble matter not removed during the pretreatment step. The COD load was highly variable from the time of collection (from 0.6 to 2.8 g-COD L^-1^). This allowed us to study the response of the ME-FBR to different organic loads when the biomass was already adapted without the need of dilution or addition of external an organic source to the real wastewater. Due to the inoculum in the ME-FBR and the broad range of energy substrates, diverse anaerobic-based metabolisms were likely to coexist. Therefore, the organic matter (mainly soluble) in the ME-FBR might be degraded throughout a serial of anaerobic biological reactions including fermentations (complex organic matter like sugars and proteins), acidogenesis (further complex matter degradation to short chain acids, H_2_, alcohols, ammonia…), acetogenesis, methanogenesis and electrogenesis (**Figure 1**). These three last pathways may compete for the acids and H_2_ (electron sources) coming from the acidogenesis step. The brewery effluent fed to the ME-FBR contained as possible main electron acceptors a fluidized anode and carbon dioxide. This represents a niche for methane-producing microorganisms, acetate-synthesizing microbes, and electroactive bacteria that are able to harvest energy from the reduction of those substrates.

### ME-FBR for Treating Organic Matter and VFAs Degradation Tests

The ME-FBR could effectively degrade the organic matter from the brewery effluent over a wide range of influent organic loads (Supplementary Figure 2). Actually, as the ORL in the ME-FBR increased, the removal of organic matter followed the same trend, reaching values always over 74%.

No VFA accumulation was observed during the experimental period, although the levels of these species increased with the increase of the OLR (Supplementary Figure 3). The levels of acids were always low (below 35 mg L^-1^ of total VFAs), revealing that the bio-electrochemical system efficiently oxidized most of the acids formed from the complex organic substrates. Actually, these acids were far below the concentrations reported to inhibit the methanogenic community in anaerobic digesters (can start from 900 mg L^-1^ of propionic acid at levels of 2400 mg L^-1^ of acetic acid) (Wang et al., 2009). Acetic acid, the main contributor to methane generation, was indeed the most abundant acid in the effluents (up to 28 mg L^-1^). Butyric acid was detected as well but at lower concentrations. Although the brewery wastewater already contained propionic acid (Supplementary Table 1), it could not be detected in the effluent of the reactor, indicating that it was rapidly consumed or not produced as much as acetic or butyric.

**FIGURE 2:**
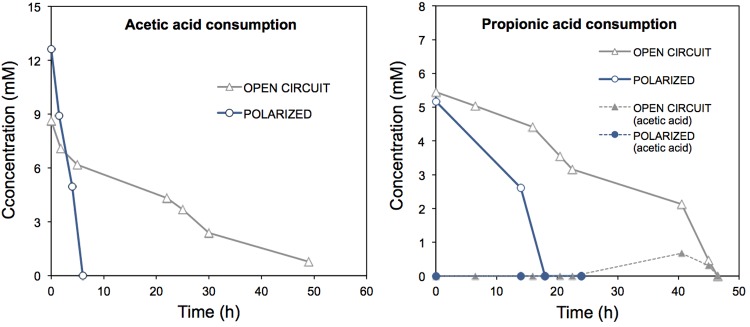
Consumption of acetic acid and propionic acid in the ME-FBR operated under batch mode, under both open circuit (fluidized anode can not act as electron acceptor) and polarized anode conditions (fluidized anode acting as electron acceptor, *E* = 0.2 V vs. Ag/AgCl). Propionate consumption led to acetic acid production, which is represented as well in the propionic acid consumption graph.

In order to further analyze the oxidation of the acids in the ME-FBR, we analyzed the individual consumption of acetate and propionate. These two acids have been reported as preferential acids by electroactive bacteria over others with higher chain lengths (Freguia et al., 2010). The VFA degradation tests were assayed by providing either the fluidized anode as final electron acceptor (electrolysis mode), or the fluidized bed as a mere carrier at open circuit (no current flow). Interestingly, both acids were consumed notably faster (**Figure 2**) under bio-electrochemical conditions (removal rates of *ca.* 35 μmol-acetate min^-1^ and 4.8 μmol-propionate min^-1^) than under OC conditions (*ca.* 2.7 μmol-acetate min^-1^ and 2 μmol-propionate min^-1^). Under the electrolysis mode, the removal rates of acetate and propionate per working reactor volume were of 5.4 g-COD L^-1^_NRV_ d^-1^and 1.3 g-COD L^-1^_NRV_ d^-1^, respectively. However, at OC the rates were of 0.4 g-COD L^-1^_NRV_ and 0.54 g-COD L^-1^_NRV_, respectively. In contrast with the high enhancement of acetate removal (13-fold), propionate removal rate was enhanced only by 2.4-fold when the fluidized bed acted as electron acceptor. The removal rates in the ME-FBR described for other METs with flat-electrode designs (Freguia et al., 2010). This rapid VFA consumption in our fluidized anode could represent an attractive tool for stabilizing anaerobic digestion processes when inhibitory processes occur that lead to acid accumulation, as previously described (Cerrillo et al., 2016).

The current response to acetate addition was inmediately detected, whereas for propionate pulse the increase in current production was not as sharp and rapid (Supplementary Figure 4). This suggests that acetate was a ready-to-use substrate for electroactive bacteria in the ME-FBR, but propionate had to be fermented first (to acetate or formate) in order to be bioelectrochemically oxidized, as previous studies have observed (Hari et al., 2016). We observed that current was still produced at the fluidized anode even when no acids were detected in the medium of the ME-FBR. This could have been due to a possible adsorption of these acids within the biofilm and on the surface of the particles.

The coulombic efficiencies (CE) values obtained were low (38% for acetate and 5% for propionate). These numbers suggest the presence of non-electrogenic microorganisms consuming those acids. These could be acetothrophic methanogens or syntrophic propionate-oxidizing bacteria leading to methane production (Kouzuma et al., 2015). Actually, methane was detected in the headspace of the reactor after an acid pulse, but not quantified (data not shown).

### Influence of the OLR Over the Bioelectrochemical Performance

**FIGURE 3:**
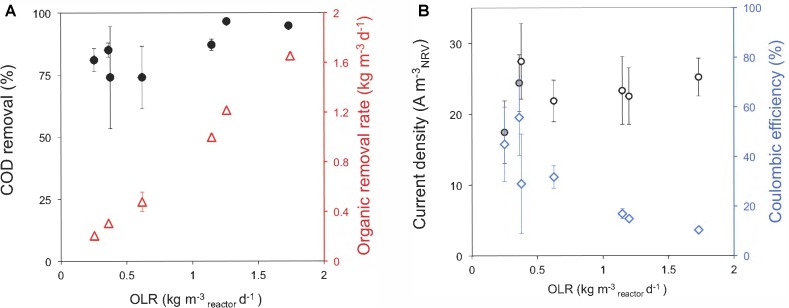
Performance of the ME-FBR on the treatment of a brewery effluent. **(A)** Chemical oxygen demand (COD) removal of the system and organic removal rates (ORRs) for all the organic loading rates tested (OLRs). **(B)** Current density harvested in the fluidized anode and coulombic efficiencies for each OLR tested in the ME-FBR. The fluidized bed was polarized to 0.2 V (vs. Ag/AgCl).

Next, we performed a serial of assays for testing the treatment capacity of the reactors at different OLRs. Our results showed that the removal efficiency significantly rise with higher OLRs within the range from 0.62 to 1.26 kg m^-3^_NRV_ d^-1^ (**Figure 3A**) (statistical analysis shown in Supplementary Table 3). Meanwhile, the organic removal rate (ORR) increased linearly with the OLR over the ranges applied. By operating the ME-FBR at higher OLRs than 1.26 kg m^-3^_NRV_ d^-1^, like 1.7 kg-COD m^-3^_NRV_ d^-1^, we did not achieve better COD removal efficiencies. In contrast, it was possible to obtain higher values of removal rates. This indicates that our configuration could further operate at ORLs higher than 1.7 kg-COD m^-3^_NRV_ d^-1^ while maintaining performance levels of 95% of COD removal.

Regarding the current density values, our ME-FBR achieved values from 17 ± 4 to 27 ± 5 A m^-3^_NRV_ (*ca.* 131 ± 33 to 206 ± 40 A m^-3^_bed_). As the OLR in the ME-FBR increased, lower values of CE were achieved. This actually occurred in spite of obtaining higher current density values at increasing OLRs for most of the conditions assayed (**Figure 3B**). The increase in current density was significant at lower OLRs (Supplementary Table 2 and statistical analysis shown in Supplementary Table 3). This effect has been extensively observed in bio-electrochemical systems with mixed cultures, and it seems to be due to the out-competition of other microbial metabolisms, as methanogenesis, at high organic loadings (Sleutels et al., 2016). Another possibility is that the existence of DIET (either via electrode-mediation or by direct cell contact) might be another cause minimizing electron recovery at the fluidized anode (Liu et al., 2012; Shrestha et al., 2013).

**FIGURE 4:**
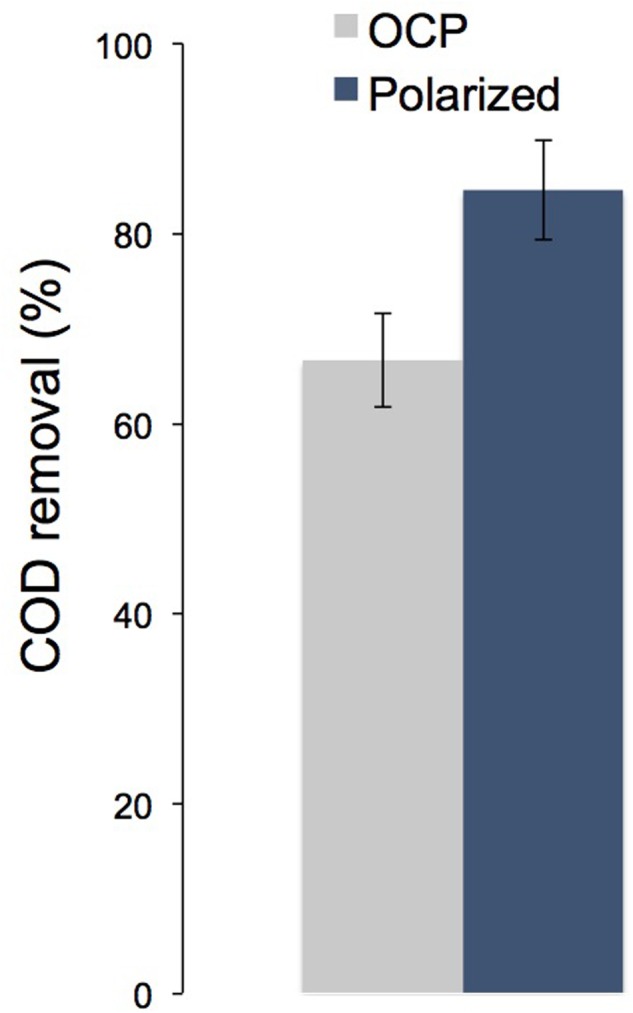
Chemical oxygen demand removal when the ME-FBR was operated under open circuit conditions and under current-circulating conditions (fluidized bed polarized to 0.4 V vs. Ag/AgCl) (OLR = 1.46 ± 0.2 kg COD m^-3^_reactor_ d^-1^).

When we ran the system under open circuit conditions at an OLR of 1.46 ± 0.2 kg COD m^-3^_NRV_ d^-1^ and then was operated back with the fluidized bed polarized, we saw an increase in COD removal of a 17% (**Figure 4**). This indicates that the electrodes polarization (current production) had a real impact in the organic matter removal.

The additional control, the biolite M-FBR (non-bioelectrochemical configuration) showed a different response to the variation of the OLR. This reactor showed much lower treatment efficiencies than the ME-FBR when operated under identical conditions (Supplementary Figure 5). The removal of COD in this reactor severally decreased when either the OLR or the flow rate were increased, which means that the system was being overloaded. This difference could be associated with the double role of the electrically conductive bed: (a) for attachment, (the chemical nature of the bed may influence bacteria attachment and biofilm development) and (b) as the electron accepting element (the bed may act as a respiratory substrate by accepting electrons from cell metabolism). Biolite has been reported as a highly biocompatible material that promotes high biomass adhesion, and rapid start-up periods during the treatment of industrial wastewaters (Balaguer et al., 1997). For this reason, we hypothesize that the nature of the material was not limiting biofilm development and biomass acclimation. Probably, the presence of an unlimited electron acceptor (polarized fluidized anode) caused the enhancement in the microbial colonization of the anodic particles.

After testing the influence of the OLR over the ME-FBR removal capacity, we analyzed the effect of eliminating the motion of the polarized particles. When the recirculating pump was switched off, the system becomes a fixed bed reactor. The performance of the reactor severally decreased from an average of 87 ± 5% of COD removal when the bed was fluidized to a minimum of 36% when the bed was fixed (Supplementary Figure 6). Under this last condition, fresh substrates, metabolites and end products are slowly transported across the biofilm and bed particles. Furthermore, at any extracelullar electron acceptor respiration, e.g., an anode, the reaction is typically consuming electrons but not protons, so this charge uncoupling may cause an acidification of the biofilm as a result of the bioelectrochemical oxidation of organic matter (Torres et al., 2008). Eventually it would lead to a depletion of the electroactive activity of the community. However, the recirculation flow of the ME-FBR can play a key role in avoiding such a biofilm acidification. Indeed, this could be one of the reasons for which eliminating the fluidization state of the bed negatively affected the ME-FBR.

In this study, we show the treatment of a brewery wastewater in a ME-FBR as a proof of concept. However, for developing a scalable prototype, each of the elements of design (the electrode material and active surface, distance between anode and cathode, recirculating flow, bed quantity, particle size or pH-control) should be properly studied and optimized. To compete on a commercial scale with high-rate anaerobic reactor configurations, such as UASB (Upflow Anaerobic Sludge Blanket), EGSB (Expanded Granular Sludge Bed), or fluidized bed configurations, which can already treat up to 40 kg-COD m^-3^_NRV_ d^-1^ (van Lier et al., 2016), the OLRs in the ME-FBR should be considerably enhanced. However, under this scenario METs so far face the problem of low electron recovery efficiencies. This minimizes the revenues (recovery of hydrogen or other by-products from cathodic reactions) that can be subtracted from bio-electrochemical treatment.

Thus, until METs are able to show higher reaction efficiencies, ME-FBRs and other bio-electrochemical-based configurations are confined to specific applications in which anaerobic reactors experience operational problems. Likewise, METs can find use as a complementary technology for further reducing the COD of the effluents coming from a first biological step. In fact, our ME-FBR has shown good bio-electrochemical behavior at low OLRs, achieving CE values up to 55 ± 15% treating a real effluent.

### Microscopic Biofilm Examination

The biocatalysis in the ME-FBR is mainly performed by the biofilm colonizing the electroconductive bed. As such, a deep analysis of the surface of the bed particles by SEM and FISH provided further insights into the physical distribution of the microbial community.

**FIGURE 5:**
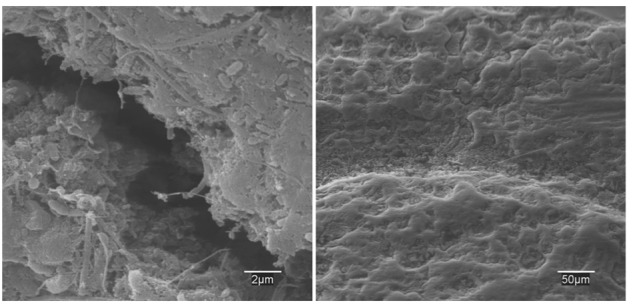
Scanning electron microscopy (SEM) images of the colonization on the particles of the ME-FBR after 4 months of operation.

We observed microbial colonization of the activated carbon particles in big pores (**Figure 5**). The biofilm developed a high content of EPS and matrix, which is typical from systems exposed to shearing stress in fluidized beds. It has been previously observed that the thickness, structure and even the metabolism of fluidized-bed biofilms are highly correlated to the detachment force (Chang et al., 1991; Liu and Tay, 2001). Thus, the limited thickness of the biofilm on the polarized particles could be a consequence of high shear conditions in the ME-FBR.

Regarding the colonization of the biolite particles of the M-FBR, we observed a poor microbial attachment after *ca*. 2.5 months of operation (Supplementary Figure 5). Rather than forming a biofilm, cells seemed to be attached forming aggregates. The difference between the biomass growths on the polarized fluidized anode and on the non-electrically conductive particles could be the reason for the variation in treatment efficiencies found between the two reactors.

**FIGURE 6:**
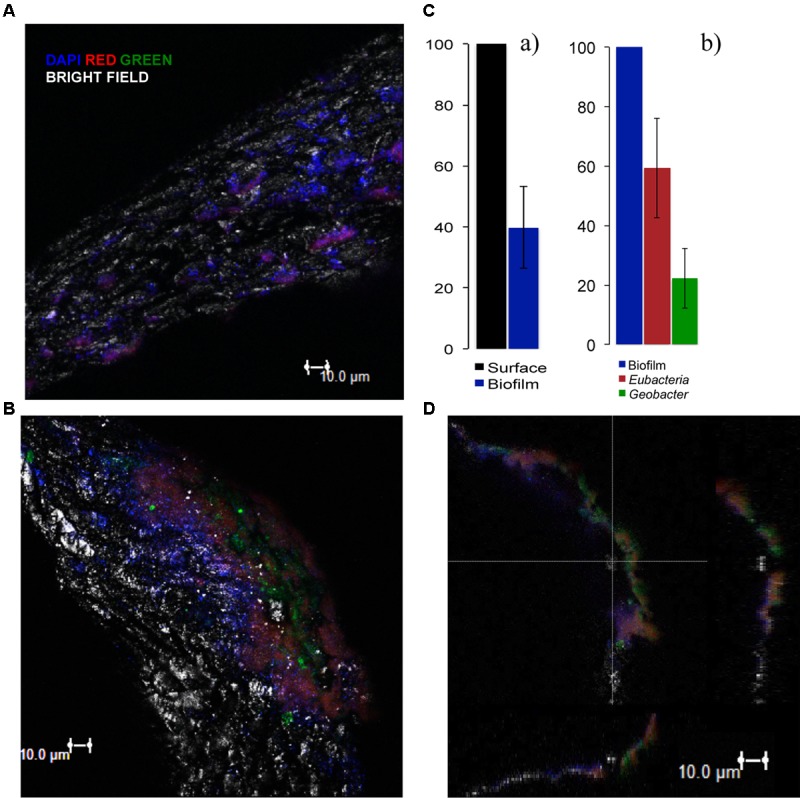
Fluorescence *in situ* hybridization (FISH) results on the polarized particles using DAPI (blue signal, all nucleic acids) and the following probes: for **(A)** Eubacteria probe (red signal) and *Geobacter* cluster probe (green signal) (probes combination 4) and for **(B)** Eubacteria (green signal) and Archaea probe (red signal) (probes combination 5). **(A,B)** 3D Images projected from a sequence of images (sections were taken at 2-μm interval) of the biofilm formed over the surface of the particles (bright field). **(C)** (a) Biomass coverage of the fluidized particles (average of 2 independent projected sequences) and (b) relative abundance of *Eubacteria* and *Geobacter* cluster estimated from at least 2 sequences of images taken for each sample (average of 3 independent projected sequences). **(D)** Sections of the biofilm from image **B** in the 3 dimensions. The 3D images were built by the software from projected sequences of 18 images, each one with an interval of 2 μm.

We further characterized the electroactive biofilm on the activated carbon particles of the ME-FBR by FISH analyses. The results with the probes and the DAPI staining showed a partial coverage with biomass of the surface of the particles (**Figure 6A**). The estimation from the DAPI staining showed that an average of *ca.* 40% of the surface (shown on the images with the bright field) was colonized with biofilm (**Figure 6C**). The existence of bare spaces without an attached biomass may actually favor the electrical connection among the fluidized particles and between the particles and current collector.

From the fluorescence images (Supplementary Figures 7, 8) we could estimate the relative abundance of each hybridized probe. Almost 60 ± 17% of the total biomass was composed of Eubacteria domain. Interestingly, the images showed a relative high abundance of *Geobacter* species (green) in the biofilm of the polarized particles (*ca*. 20 ± 10%) (**Figure 6C**). The presence of this genera characterized by performing efficient direct EET suggests a strong role of *Geobacter* for oxidizing VFAs and donating the resulting electrons to the fluidized anode (Aklujkar et al., 2009). It also indicates that the polarization of the conductive particles highly determined the microbial diversity in the biofilm and its structure.

Similar observations have been described in other studies with METs treating real wastewaters from the food industry, in which the anode presents a high proportion of *Geobacter* species (Kiely et al., 2011; Blanchet et al., 2015).

The analysis with the Archaea probe revealed the important presence of species of this domain (**Figures 6B,D**). An estimated average of *ca.* 50% of Archaea domain from the total biomass was obtained (see Supplementary Figure 8). This could be explained by the fact that the particle samples were collected when the ME-FBR was operated at the highest OLR (1.7 kg-COD m^-3^_NRV_ d^-1^). This condition probably stimulated the growth of methanogens.

### Microbial Stratification Within the Biofilm

**FIGURE 7:**
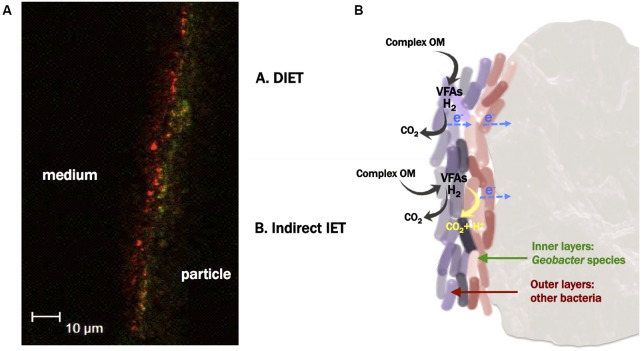
**(A)** FISH image from the section of the biofilm developed on an activated carbon particle, using *Eubacteria* probe (red signal), and *Geobacter* cluster probe (green signal) (probes combination 3). The left side of the red signal corresponds to the outermost environment (ME-FBR medium), whereas the right side of the green signal corresponds to the surface of a fluidized activated carbon particle. This image is representative of one out of 30 sections taken from the biofilm developed on a surface of *ca*. 50 × 100 μm. **(B)** Proposed microbial electron transfer mechanisms among the different microbial communities colonizing the particles and toward the fluidized anode.

The images taken with the confocal microscope allowed us to visualize a biofilm thickness of *ca.* 10 μm (**Figures 6D**, **7A**). This thickness is relatively low in comparison with either previously reported anodic biofilm (Jana et al., 2014) or with non-electroactive fluidized bed biofilms (Liu and Tay, 2001). Probably the fluidized particles in the ME-FBR were exposed to a high shearing stress that could be limiting the biofilm growth.

Hybridization using both Archaea and Eubacteria probes did not show a clear organization of these two microbial communities (**Figure 6D**). Nevertheless, we observed a pronounced microbial stratification of the biofilm when the *Geobacter* cluster and Eubacteria probes were employed (probes combination 3). The most internal layers of the biofilm were mainly composed of bacteria from the *Geobacter* genus, whereas at the outermost zones of the film other kinds of bacteria were present (**Figure 7A**). This strongly suggests that members of the *Geobacter* genus could be responsible for the direct and ultimate transfer of electrons to the anode in the ME-FBR. These electrons may be generated from *Geobacter* metabolism, or from other microorganisms able to perform interspecies electron transfer (Shrestha and Rotaru, 2014). It has already been reported through gene sequencing that the *Geobacter* genus dominates over other microbial communities at the most internal layers of current-producing mixed-species biofilms (Malvankar et al., 2012). However, we show for the first time images of this microbial stratification on an anodic biofilm. This scenario was observed again when the *Geobacter* cluster probe was used in combination only with DAPI (probes combination 6, see Supplementary Figure 10A). Indeed, a higher presence of bacteria domain at the internal layers of the biofilm was observed (probes combination 4, Supplementary Figure 10B).

This microbial stratification and *Geobacter* selection might have been favored by the initial enrichment strategy used for starting-up the ME-FBR (acetate was added to the ME-FBR). This might have stimulated an initial *Geobacter* colonization of the particles surface. The brewery wastewater initially contained VFAs such as acetate and propionate, which are also generated during the fermentation processes in the ME-FBR together with H_2_. Thus, it is likely that these conditions promoted as well an additional *Geobacter* enrichment on the particles (secondary biofilm development).

While in the case of anode-reducing biofilms a clear microbial conformation (Kiely et al., 2011) has not been identified, a defined stratification was found on electrode-oxidizing biofilms by using fluorescence *in situ* hybridization (FISH) (Virdis et al., 2011). In this study, electroactive denitrifying microorganisms using a cathode as electron donor were located at the inner layers of the biofilm on the electrode, whereas ammonia-oxidizing species were found in the outermost layers of the film. This microbial stratification was due to the existence of an oxygen gradient within the biofilm.

The scenario found in our anodic fluidized particles suggests the existence of several kinds of microbial interactions within the biofilm. We hypothesize two mechanisms that may act independently or coexist, through either direct or indirect interspecies electron transfer (DIET vs. IIET, see **Figure 7B**). The first hypothesis is that members of the *Geobacter* genus could be responsible for taking up the electrons from the metabolism of other species, acting like a biological *plug* between outermost cells and the fluidized anode surface by performing DIET (mechanism shown as case A in **Figure 7B**) (Lovley, 2011; Shrestha et al., 2013). Thus, this direct electron transfer might be directional, from the outer to the innermost layers of the biofilm. This mechanism would allow to indirectly recovering of electrical current from complex substrates that are not substrates for *Geobacter (such as sugars)*.

Previous studies have described the stimulation of DIET between bacteria and methanogens in the presence of granular activated carbon (GAC), without any polarization of this material (Liu et al., 2012). In that study, the mere electrically conductive nature of GAC promoted the exchange of electrons without the need of cell aggregation as typically occurs when performing DIET. However, no stratification was observed within that biofilm, which suggests that the polarization value (e.g., 0.2 V versus Ag/AgCl) of our fluidized activated carbon particles may have determined preferential directions for the electron flux, from the outermost layers of the biofilm to the anode. The cell assemblage along the biofilm thickness might be a consequence of the resulting redox gradient.

It remains unknown whether the operation of the ME-FBR at open circuit potential for a long period of time would have led to a different microbial conformation within the biofilm. Further investigation regarding this approach might provide interesting information concerning strategies, which might be employed for interspecies electron transfer.

Our second hypothesis is that the assemblage of *Geobacter* cells at the inner layers of the polarized fluidized particles could be directly metabolizing intermediate metabolites that reach probably by diffusion the deeper layers of the biofilm (Lovley, 2011) (this mechanisms is shown as case B in **Figure 7B**). Under this scenario, the communities would be sharing metabolites as energy currency, and cooperating to degrade the organic substrates contained in the brewery effluent. Our ME-FBR reactor was fed by brewery wastewater rich in complex substrates that were used by fermenters and converted into smaller organic molecules like VFAs. Thus, synthrophic metabolic relations were likely to occur among the different microbial communities. Bacteria from *Geobacter* genus have been reported to be capable of oxidizing a wide variety of short-chain acids, like acetate (Bond and Lovley, 2003), lactate (Call and Logan, 2011; Speers and Reguera, 2012), formate (Speers and Reguera, 2012) and directly transferring the resulting electrons to an anode. In addition, hydrogen, which is an end-product from the metabolism of acetogens and an electron shuttle in interspecies electron transfer (McInerney et al., 2009; Stams and Plugge, 2009), can also be used as electron donor to *Geobacter* (Bond and Lovley, 2003).

This second scenario has been described as mediated interspecies electron transfer via soluble metabolites (Rotaru et al., 2012; Shrestha and Rotaru, 2014). The outer-*Geobacter* cells within the biofilm might be provided by fresh and ready-to-use non-fermentable substrates. Those cells, not in intimate contact with the anode, may use long-range electron transfer strategies to reach the fluidized anode, like cell-to-cell or cell-to-electrode electron transfer via electrically conductive nanowires as has been described for insoluble electron acceptors (Reguera et al., 2005; Strycharz-Glaven et al., 2011). In contrast, the first layer of cells might be respiring the anode by directly contacting the electrode surface via *c*-type cytochromes (Busalmen et al., 2008).

As previously suggested, both mechanism proposed in **Figure 7B** may coexist, prevailing one over the other depending upon the electron donor available, and the distance to the anode.

## Conclusion

In this study, we expand the classical use of static electrodes for performing microbial electrochemistry by demonstrating that a fluidized anode made of electrically conductive particles is a suitable configuration for treating a real industrial wastewater such as a brewery effluent. The design of our ME-FBR was able to remove COD from wastewater at a rate further than an OLR of 1.7 kg-COD m^-3^_NRV_ d^-1^. Our results showed that the proportion of electrogenic metabolism highly depended on the OLR applied. Increasing the OLRs in the ME-FBR lead to a decrease in electron recovery on the fluidized anode. Thus, one should examine methods to stimulate the degradation of simple organic matter by microbial electrogenesis rather than by other metabolisms.

Working with the fluidized anode serving as electron acceptor (polarization conditions) resulted in improved organic matterremoval rates. Furthermore, we observed that the nature of the fluidized bed (electrically or non-electrically conductive) highly influenced the treatment efficiency and the microbial colonization of the reactor.

The microbial analysis revealed the development of a thin biofilm on the fluidized particles of the ME-FBR. A microbial stratification was observed in it, in which *Geobacter* was naturally located at the inner layers of the biofilm, in intimate contact with the fluidized anode. This scenario shows the existence of a microbial organization in space created by the presence of an extracellular electron acceptor like a fluidized anode. The fact that *Geobacter* species are stacked immediately adjacent to most internal layers shows the competitive advantage of this species over others for respiring at an anode, and the relevant role of these *Geobacter* species on interspecies electron transfer. Further studies regarding the factors that promote this microbial distribution should be addressed in order to better understand the role of *Geobacter* in natural environments where interspecies electron transfer is a key survival strategy for energy conservation.

## Author Contributions

ST-S designed the study, carried out the majority of the experiments and draft the manuscript. PF-L and SH helped with the experimental work. AE-N and CT supervised the study and corrected the manuscript. All authors read and approved the final manuscript.

## Conflict of Interest Statement

The authors declare that the research was conducted in the absence of any commercial or financial relationships that could be construed as a potential conflict of interest.
