# Supplementary material for: Geobacter Dominates the Inner Layers of a Stratified Biofilm on a Fluidized Anode During Brewery Wastewater Treatment
Source: Front Microbiol. 2018 Mar 6;9:378. doi: 10.3389/fmicb.2018.00378 (PMC5853052; doi:10.3389/fmicb.2018.00378)
Supplement: Supplementary file 1 [file Table_1.DOCX]

**Supplementary Table 1:** Characteristics of the brewery wastewater collected at the beginning of the study. COD values are given as an interval as each collected batch of wastewater presented a different organic load.

| **Parameter** | **Value** |
| --- | --- |
| Total COD (g L^-1^) | 0.6-2.8 |
| TOC (g L^-1^) | 0.7 |
| VFAs (g L^-1^) | 1 |
| Acetic acid (g L^-1^) | 0.5 |
| Propionic acid (g L^-1^) | 0.4 |
| Butyric acid (g L^-1^) | 0.03 |
| Conductivity (mS cm^-1^) | 3.5 |
| pH | 7 |
| TSS (mg L^-1^) | 55.4 |
| Total N (mg L^-1^) | 25 |
| NH_4_^+^ | 9.5 |
| NO_2_^-^ (mg L^-1^) | n.d |
| NO_3_^-^ (mg L^-1^) | n.d |
| Total P (mg L^-1^) | 6.8 |
| Inorganic carbon (mg L^-1^) | 135 |
| Alkalinity (mg L^-1^CaCO_3_) | 1.195 |
| Turbidity (NFU) | 388 |

n.d.: not detected
