# Supplementary material for: Geobacter Dominates the Inner Layers of a Stratified Biofilm on a Fluidized Anode During Brewery Wastewater Treatment
Source: Front Microbiol. 2018 Mar 6;9:378. doi: 10.3389/fmicb.2018.00378 (PMC5853052; doi:10.3389/fmicb.2018.00378)
Supplement: Supplementary file 2 [file Table_2.DOCX]

**Supplementary Table 2:** Operating conditions and performance of the ME-FBR at each assay.

| **Influent flow rate**  **(mL d^-1^)** | **HRT**  **(days)** | **Organic loading rate (kg-COD m^-3^ d^-1^)** | **COD effluent**  **(mg L^-1^)** | **COD removal (%)** | **Coulombic efficiency (%)** |
| --- | --- | --- | --- | --- | --- |
| 0.25 | 2.4 | 0.36 | 130 ± 26 | 85 ± 3 | 53 ± 15 |
| 0.25 | 2.4 | 0.62 | 370 ± 150 | 74 ± 13 | 32 ± 5 |
| 0.25 | 2.4 | 0.38 | 235 ± 52 | 74 ± 21 | 29 ± 20 |
| 0.25 | 2.4 | 0.25 | 112 ± 51 | 81 ± 5 | 45 ± 15 |
| 0.25 | 2.4 | 1.15 | 365 ± 64 | 87 ± 2 | 17 ± 2 |
| 0.47 | 1.28 | 1.26 | 56 ± 18 | 97 ± 1 | 15 ± 1 |
| 0.62 | 0.98 | 1.73 | 88 ± 23 | 95 ± 1 | 10 ± 1 |
