# Supplementary material for: Geobacter Dominates the Inner Layers of a Stratified Biofilm on a Fluidized Anode During Brewery Wastewater Treatment
Source: Front Microbiol. 2018 Mar 6;9:378. doi: 10.3389/fmicb.2018.00378 (PMC5853052; doi:10.3389/fmicb.2018.00378)
Supplement: Supplementary file 3 [file Table_3.DOCX]

**Supplementary Table 3**: Statistical analysis for assessing the significance differences of COD removals and current densities achieved at the different OLRs tested in the ME-FBR (the confidence interval was of 95 % and the significance level of 0.05).

| **Compared variables** | **Student’s t** | **Probability<t** | **Significant difference** |
| --- | --- | --- | --- |
| % COD removal for  OLR of 0.25 kg m^-3^ d^-1^ and 0.36 kg m^-3^ d^-1^ | -1.162 | 0.1375 | NO |
| % COD removal for  OLR of 0.36 kg m^-3^ d^-1^ and 0.38 kg m^-3^ d^-1^ | 4.416 | 0.999 | NO |
| % COD removal for  OLR of 0.38 kg m^-3^ d^-1^ and 0.62 kg m^-3^ d^-1^ | -0.848 | 0.211 | NO |
| % COD removal for  OLR of 0.62 kg m^-3^ d^-1^ and 1.15 kg m^-3^ d^-1^ | -2.563 | 0.0159 | YES |
| % COD removal for  OLR of 1.15 kg m^-3^ d^-1^ and 1.26 kg m^-3^ d^-1^ | -9.440 | 1.342·10^-6^ | YES |
| % COD removal for  OLR of 1.26 kg m^-3^ d^-1^ and 1.74 kg m^-3^ d^-1^ | 2.530 | 0.986 | NO |
| Current density for OLR of 0.25 kg m^-3^ d^-1^ and 0.36 kg m^-3^ d^-1^ | -52.402 | 0 | YES |
| Current density for OLR of 0.36 kg m^-3^ d^-1^ and 0.38 kg m^-3^ d^-1^ | -63.437 | 0 | YES |
| Current density for OLR of 0.38 kg m^-3^ d^-1^ and 0.62 kg m^-3^ d^-1^ | 128.84 | 1 | NO |
| Current density for OLR of 0.62 kg m^-3^ d^-1^ and 1.15 kg m^-3^ d^-1^ | -26.917 | 2.238·10^-158^ | YES |
| Current density for OLR of 1.15 kg m^-3^ d^-1^ and 1.19 kg m^-3^ d^-1^ | 30.410 | 1 | NO |
| Current density for OLR of 1.26 kg m^-3^ d^-1^ and 1.74 kg m^-3^ d^-1^ | -56.502 | 0 | YES |
