# Supplementary figures and images for: Geobacter Dominates the Inner Layers of a Stratified Biofilm on a Fluidized Anode During Brewery Wastewater Treatment
Source: Front Microbiol. 2018 Mar 6;9:378. doi: 10.3389/fmicb.2018.00378 (PMC5853052; doi:10.3389/fmicb.2018.00378)

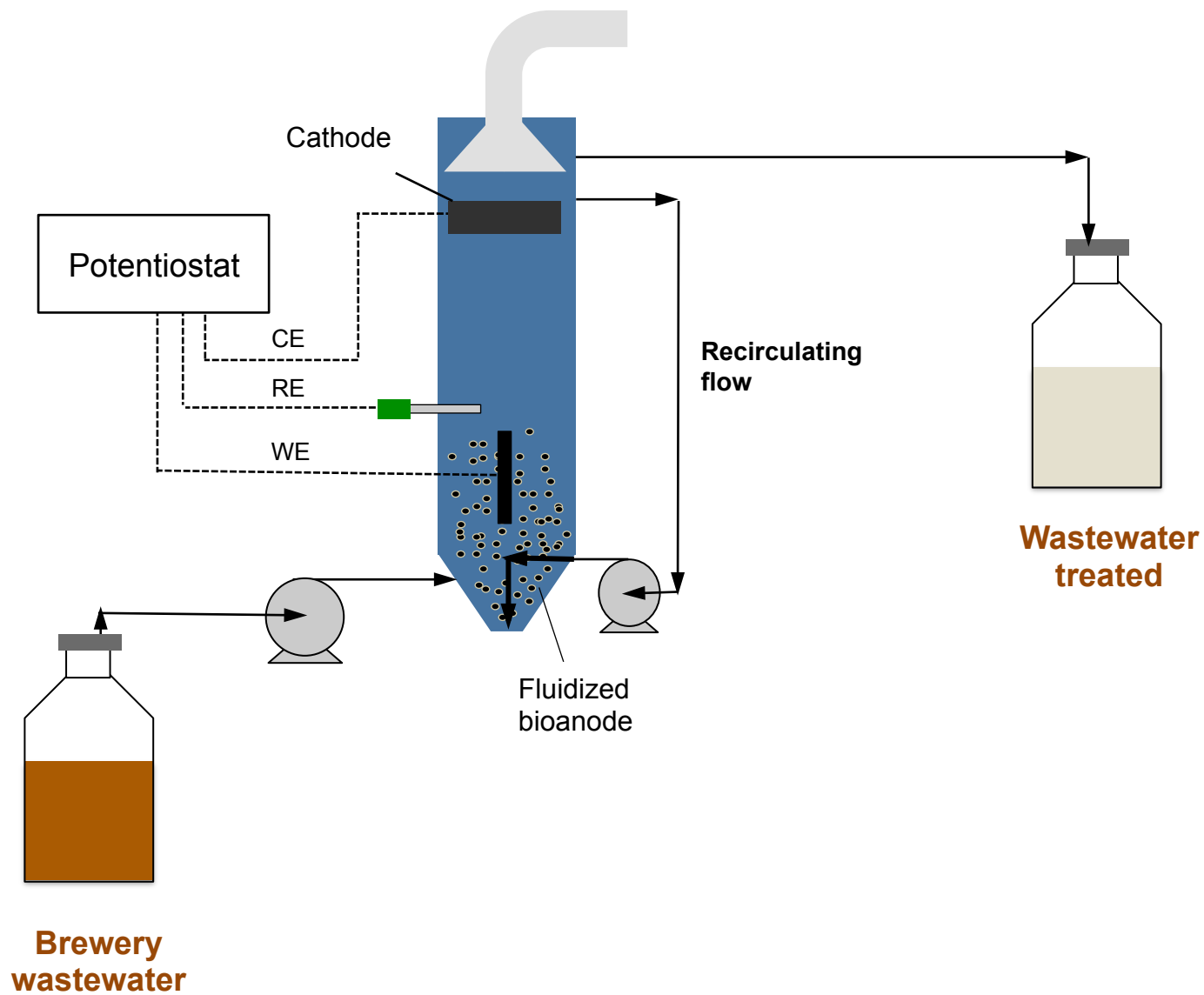

**Supplementary Figure 1:** Schematic of the operated systems at continuous mode.

Supplement: Supplementary file 4 [file Image_1.PDF]
