# Supplementary material for: Geobacter Dominates the Inner Layers of a Stratified Biofilm on a Fluidized Anode During Brewery Wastewater Treatment
Source: Front Microbiol. 2018 Mar 6;9:378. doi: 10.3389/fmicb.2018.00378 (PMC5853052; doi:10.3389/fmicb.2018.00378)
Supplement: Supplementary file 6 [file Image_3.PDF]

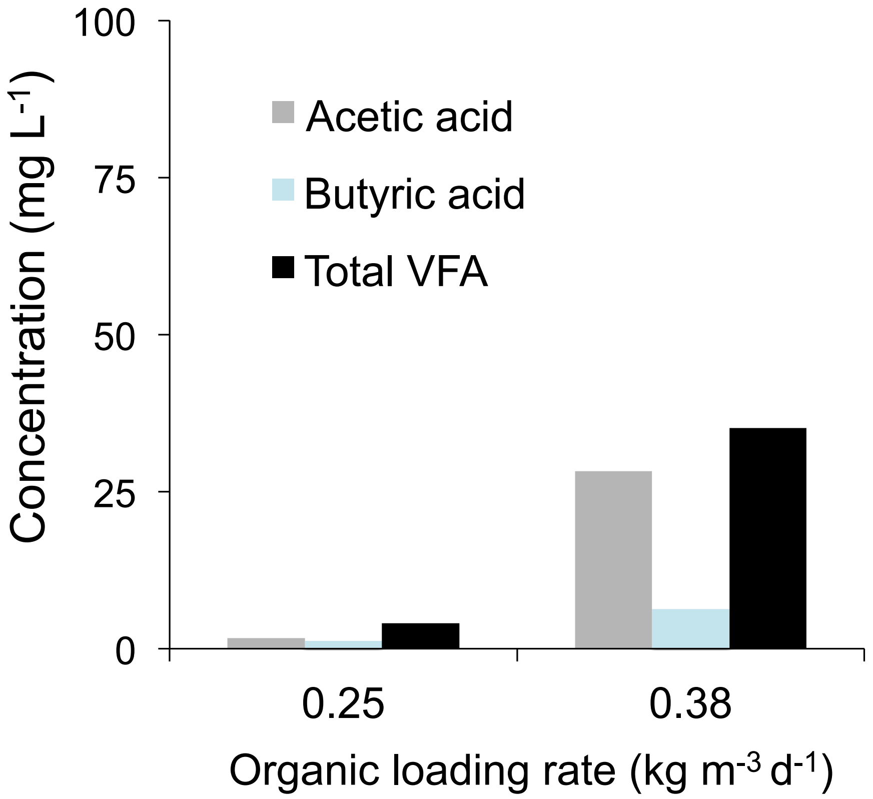

Supplementary Figure 3: Volatile fatty acids measured in the effluent of the ME-FBR at two organic loading rates (HRT of 53 h and COD of the influent of 610 and 900 mg L^{-1}).
