# Supplementary material for: Geobacter Dominates the Inner Layers of a Stratified Biofilm on a Fluidized Anode During Brewery Wastewater Treatment
Source: Front Microbiol. 2018 Mar 6;9:378. doi: 10.3389/fmicb.2018.00378 (PMC5853052; doi:10.3389/fmicb.2018.00378)
Supplement: Supplementary file 7 [file Image_4.PDF]

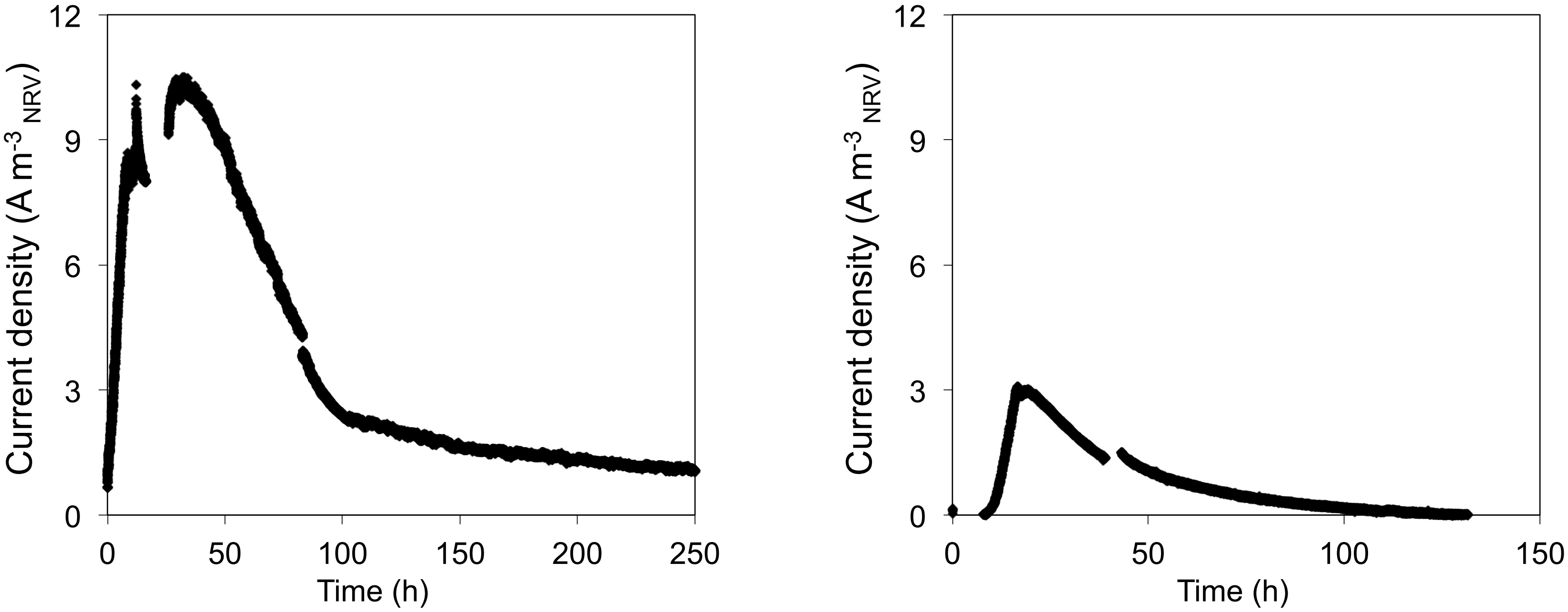

Supplementary Figure 4: Cronoamperometric assays from the acetate **(A)** and propionate **(B)** pulses. E_{anode}= 0.2 V vs Ag/AgCl.
