# Supplementary material for: Geobacter Dominates the Inner Layers of a Stratified Biofilm on a Fluidized Anode During Brewery Wastewater Treatment
Source: Front Microbiol. 2018 Mar 6;9:378. doi: 10.3389/fmicb.2018.00378 (PMC5853052; doi:10.3389/fmicb.2018.00378)
Supplement: Supplementary file 8 [file Image_5.PDF]

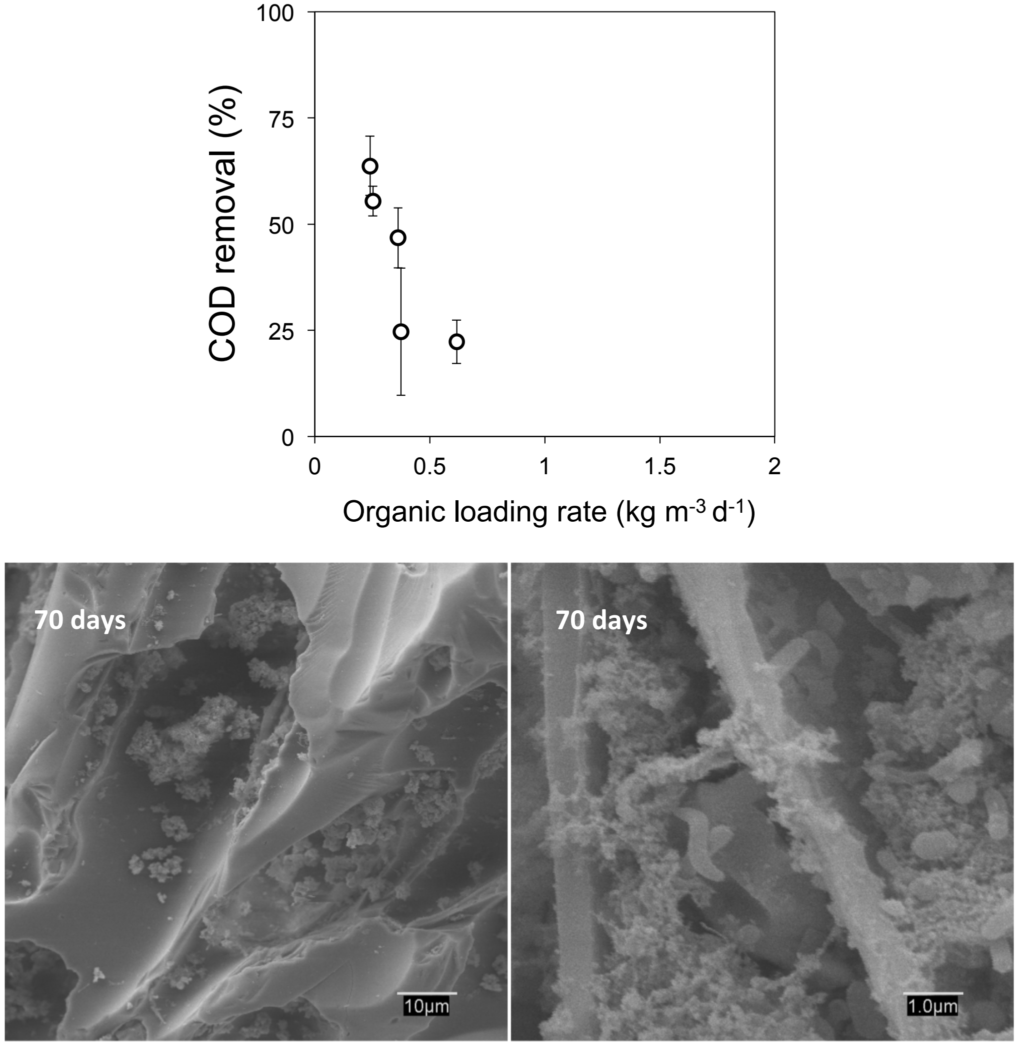


**Supplementary Figure 5:** A. COD removal of a M-FBR with sepiolite particles as bed (non conductive material) at different OLRs. B. SEM images from the sepiolite particles after 4 months of reactor operation (same time than when the ME-FBR particles were visualized).
