# Supplementary material for: Geobacter Dominates the Inner Layers of a Stratified Biofilm on a Fluidized Anode During Brewery Wastewater Treatment
Source: Front Microbiol. 2018 Mar 6;9:378. doi: 10.3389/fmicb.2018.00378 (PMC5853052; doi:10.3389/fmicb.2018.00378)
Supplement: Supplementary file 10 [file Image_7.PDF]

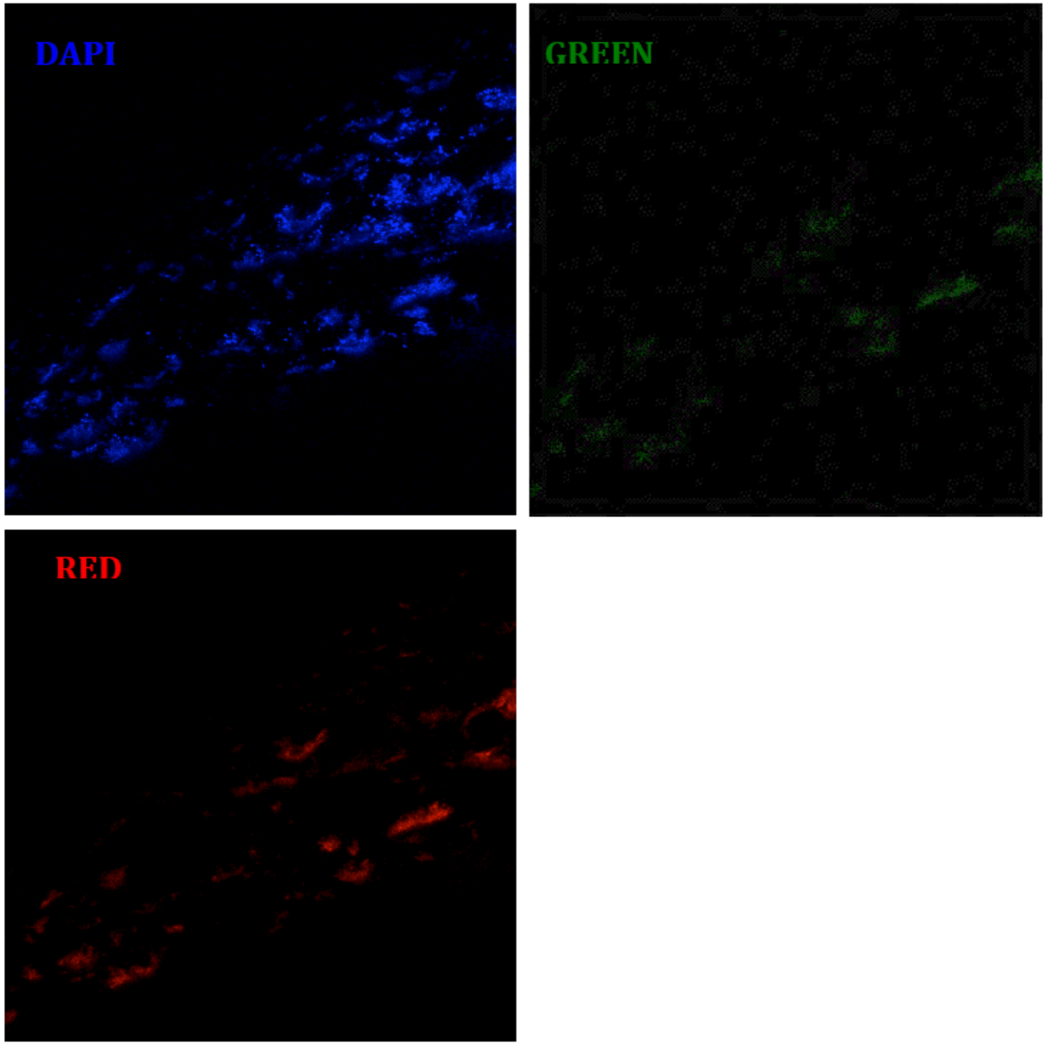

Supplementary Figure 7: 1. FISH experiments on the polarized particles of the ME-FBR. The blue signal corresponds to the DAPI stain (all nucleic acids), the red signal corresponds to the Eubacteria probe, the green one targets *Geobacter* cluster, while the white signal corresponds to the surface of the particle.
