# Supplementary material for: Geobacter Dominates the Inner Layers of a Stratified Biofilm on a Fluidized Anode During Brewery Wastewater Treatment
Source: Front Microbiol. 2018 Mar 6;9:378. doi: 10.3389/fmicb.2018.00378 (PMC5853052; doi:10.3389/fmicb.2018.00378)
Supplement: Supplementary file 11 [file Image_8.PDF]

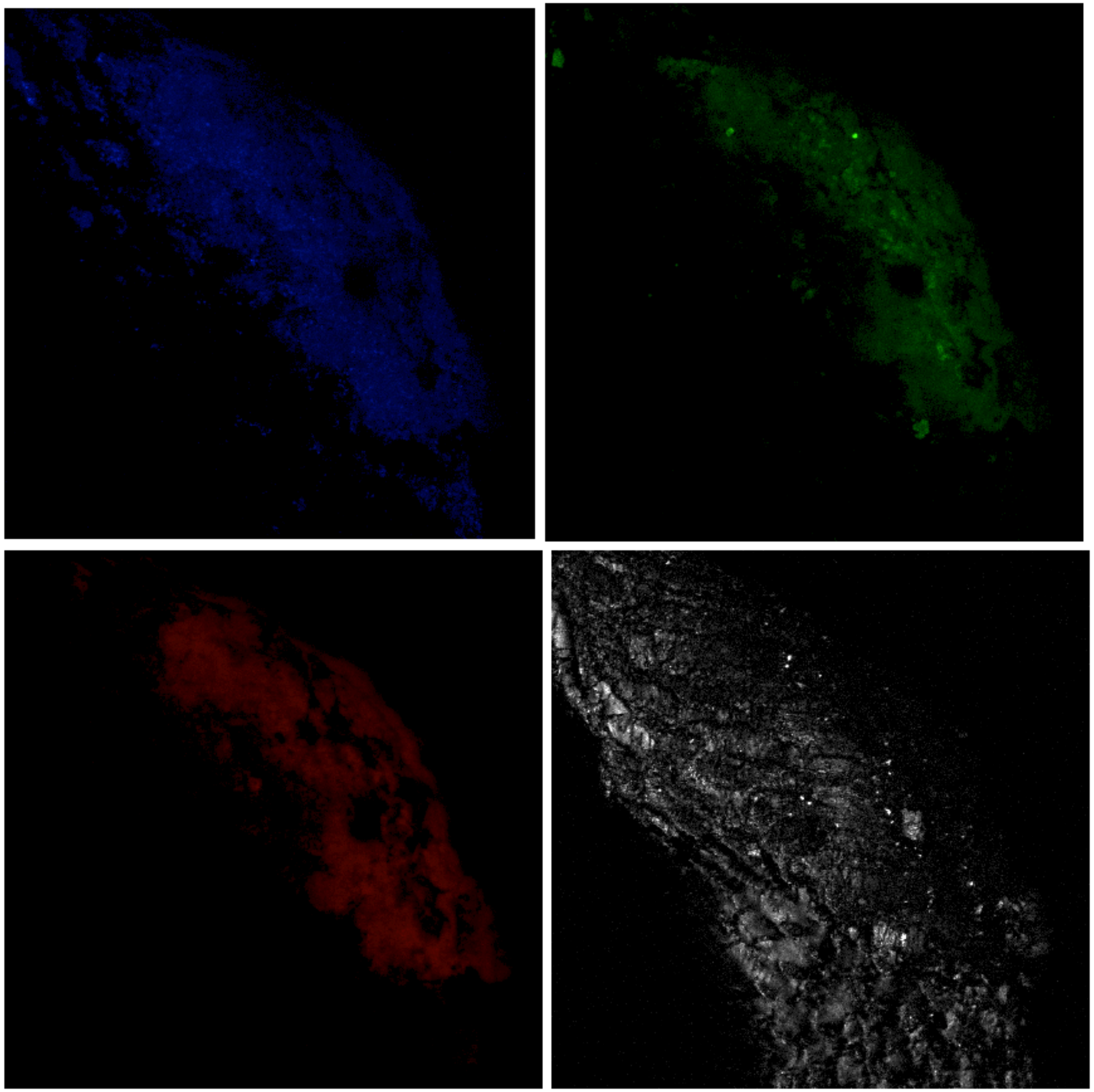

Supplementary Figure 8: 1. FISH experiments on the polarized particles of the ME-FBR. The blue signal corresponds to the DAPI stain (all nucleic acids), the green signal corresponds to the Eubacteria probe, the red one targets the Archaea, while the white signal corresponds to the surface of the particle.
