# Supplementary material for: Geobacter Dominates the Inner Layers of a Stratified Biofilm on a Fluidized Anode During Brewery Wastewater Treatment
Source: Front Microbiol. 2018 Mar 6;9:378. doi: 10.3389/fmicb.2018.00378 (PMC5853052; doi:10.3389/fmicb.2018.00378)
Supplement: Supplementary file 12 [file Image_9.PDF]

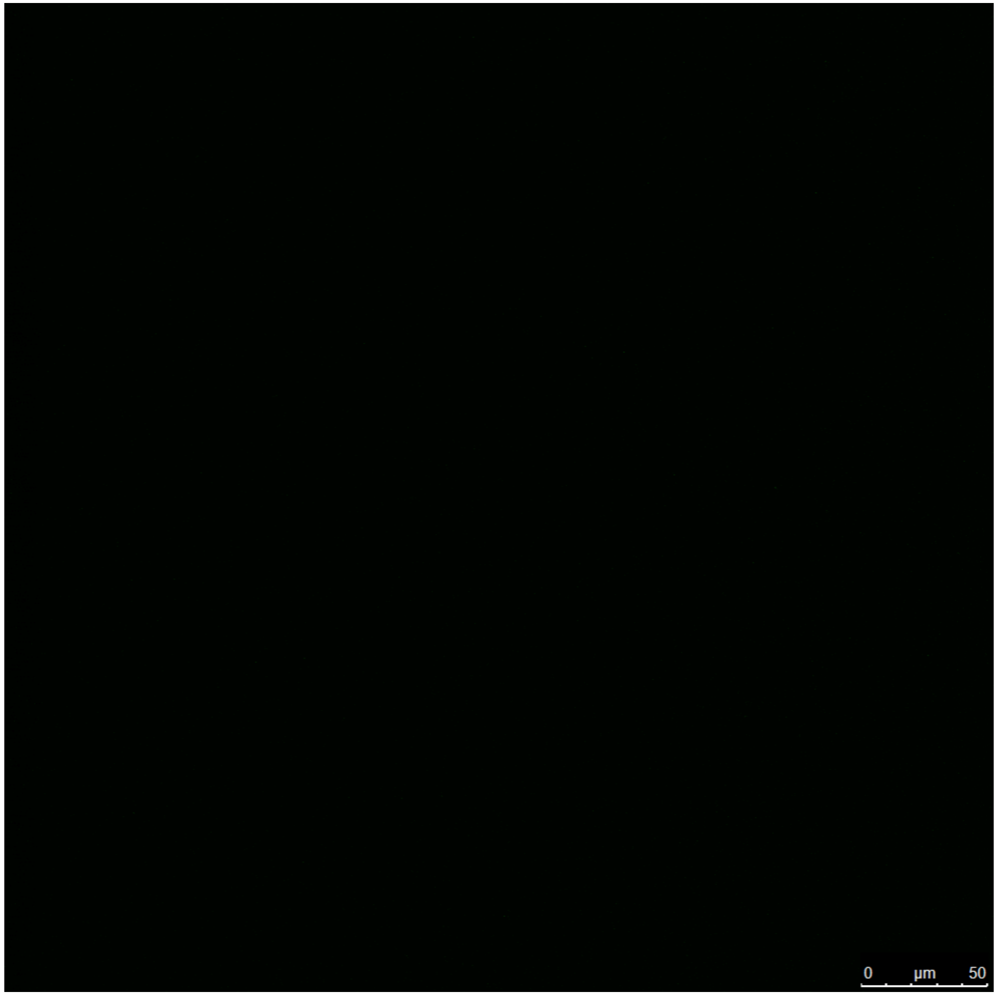

Supplementary Figure 9: Fluorescent signal with the non-specific probe on a fluidized activated carbon particle colonized with biofilm (sequence of the probe: ACTCCTACGGGAGGCAGC- Fluo).
