# Supplementary material for: Geobacter Dominates the Inner Layers of a Stratified Biofilm on a Fluidized Anode During Brewery Wastewater Treatment
Source: Front Microbiol. 2018 Mar 6;9:378. doi: 10.3389/fmicb.2018.00378 (PMC5853052; doi:10.3389/fmicb.2018.00378)
Supplement: Supplementary file 13 [file Image_10.PDF]

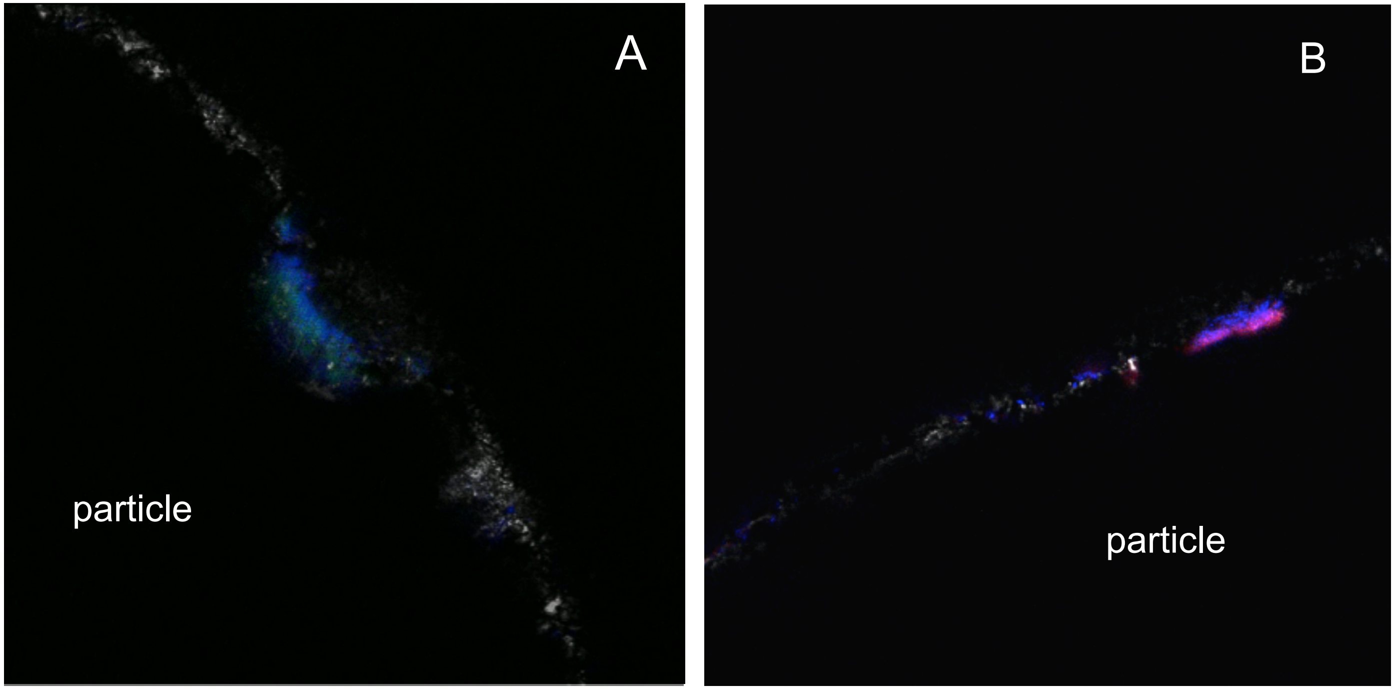

Supplementary Figure 10: Images of sections taken with a confocal microscope of the activated carbon particles used for FISH assays and that show the microbial stratification developed on them. The blue signal corresponds to the DAPI stain while the white signal corresponds to the surface of the particle. For A) signal corresponds to *Geobacter* cluster and for B) the red signal corresponds to Eubacteria.
